# Long-Term Dietary Supplementation of Pomegranates, Figs and Dates Alleviate Neuroinflammation in a Transgenic Mouse Model of Alzheimer’s Disease

**DOI:** 10.1371/journal.pone.0120964

**Published:** 2015-03-25

**Authors:** Musthafa Mohamed Essa, Selvaraju Subash, Mohammed Akbar, Samir Al-Adawi, Gilles J. Guillemin

**Affiliations:** 1 Dept of Food Science and Nutrition, College of Agriculture and Marine Sciences, Sultan Qaboos University, Muscat, Oman; 2 Ageing and Dementia Research Group, Sultan Qaboos University, Muscat, Oman; 3 Neuropharmacology group, MND and Neurodegenerative Diseases Research Centre, Macquarie University, Sydney, NSW, Australia; 4 Section of Molecular Pharmacology and Toxicology, Laboratory of Membrane Biochemistry and Biophysics, National Institute on Alcohol Abuse and Alcoholism, National Institutes of Health, Rockville, MD, United States of America; 5 College of Medicine and Health Sciences, Sultan Qaboos University, Muscat, Oman; Charité, Campus Benjamin Franklin, GERMANY

## Abstract

Alzheimer’s disease (AD) is a devastating age-related neurodegenerative disease with no specific treatment at present. The APPsw/Tg2576 mice exhibit age-related deterioration in memory and learning as well as amyloid-beta (Aβ) accumulation, and this mouse strain is considered an effective model for studying the mechanism of accelerated brain aging and senescence. The present study was aimed to investigate the beneficial effects of dietary supplements pomegranate, figs, or the dates on suppressing inflammatory cytokines in APPsw/Tg2576 mice. Changes in the plasma cytokines and Aβ, ATP, and inflammatory cytokines were investigated in the brain of transgenic mice. Significantly enhanced levels of inflammatory cytokines IL-1β, IL-2, IL-3, IL-4, IL-5, IL-6, IL-9, IL-10, TNF-α and Eotaxin activity were decreased by administration of the diet supplements containing pomegranates, figs, or dates. In addition, putative delays in the formation of senile plaques, as indicated by a decreasing tendency of brain Aβ1–40 and Aβ1–42 contents, were observed. Thus, novel results mediated by reducing inflammatory cytokines during aging may represent one mechanism by which these supplements exert their beneficial effects against neurodegenerative diseases such as AD.

## Introduction

Alzheimer’s disease (AD) is a progressive neurodegenerative disorder, characterized by abnormal accumulation of amyloid plaques and neurofibrillary tangles throughout the cerebrocortical and limbic regions [1]. Further evidence suggests that initial vascular damage plays a pivotal role in functional and structural changes of neurons [2–4], and accumulation of brain amyloid-beta (Aβ) peptides is subsequent to the blood-brain barrier (BBB) dysfunction and reduction in cerebral blood flow [3,5]. The neuropathological hallmarks of the disease are amyloid plaques and neurofibrillary tangles (NFT), which progressively accumulate in the brain. These neuropathologies are closely linked with chronic inflammation and neuronal dysfunction. Proinflammatory cytokines, such as interleukin (IL)-1β, IL-6 and tumor necrosis factor (TNF-α), have been reported to be involved in the formation of neuritic plaques in AD [6–8]. IL-1β has an important neuromodulatory role in the hippocampus, acting downstream of the initial events of long term potentiation [9]. An increased endogenous IL-1β concentration in the hippocampus may be a common trigger for impairments in long-term potentiation in age and stress-induced rats [10]. Transgenic mice over expressing IL-6 exhibited a progressive age-related decline in avoidance learning performance [11]. The Aβ plaques and NFTs accumulated in the brain activate inflammatory cells (i.e. astrocytes and microglia) and tissue levels of pro- and anti-inflammatory mediators, including cytokines and chemokines, are altered. In addition to the observation that inflammatory mediators are present in AD lesions, there is also epidemiological and genetic evidence which shows that an inflammatory process contributes to AD pathology. Prospective case-cohort studies show that higher serum levels of certain acute-phase proteins are a risk factor for the development of AD [12–15]. Certain polymorphisms of cytokines, most notably interleukin (IL)-1α, seem to be a genetic risk factor for AD [16]. Moreover, epidemiological studies indicate that longstanding use of non-steroidal anti-inflammatory drugs can prevent or retard the development of AD [17,18]. In contrast to the epidemiological studies, patients with a clinical AD syndrome do not benefit from treatment with anti-inflammatory drugs [19].

More recently, the interest in the role of dietary antioxidants in human health has prompted research in the field of AD. Fruits are good sources of these bioactive components, and there are a number of commercial polyphenol-rich beverages, which base their marketing strategies on antioxidant potency. Naturally occurring compounds from plants have been offering possible their therapeutic potential for AD [20–22].

Pomegranates (*Punica granatum Linn*.) contain very high levels of polyphenols as compared to other fruits and vegetables. The pomegranates have been extensively used in Unani, Ayurvedic and Chinese systems of oriental medicine. The plant is used in folklore medicine for the treatment of various diseases, such as ulcer, hepatic damage, snakebite, etc. Mediterranean and Middle-East countries are the main regions of pomegranate cultivation and production [23,24]. Pomegranates are a very rich source of anthocyanins (cyanidin 3,5-di and 3-O-glucoside, delphinidin 3,5- di and 3-O-glucoside, pelargonidin 3,5-di and 3-O-glucoside), ellagic acid, punicalagin isomers, different flavanols (catechins as catechins and epicatechin, and gallocatechins as gallocatechin and epigallocatechin), etc [25–27]. Dietary supplementation of pregnant mice with pomegranate juice was shown to protect against neurodegeneration in neonatal mice subjected to hypoxic–ischemic brain injury [28].

The fig (*Ficus carica L*.) is a classic fruit tree associated with the beginnings of horticulture in the Mediterranean basin [29,30]. The Mediterranean region and especially the Middle Eastern countries have been the most important center of figs growth from time immemorial [31]. Compared with other common fruits and beverages, figs are an excellent source of minerals, vitamins and dietary fiber; they are fat and cholesterol free and are contain abundant amino acids [32–35]; they also contain the highest concentrations of polyphenols [36]. The fig fruit is well known for its attractive taste, nutritive value due to its antioxidant properties, and it is consumed fresh or as dried products worldwide [37–39]. In traditional medical system, figs are used for treating various ailments including cardiovascular disease, respiratory problems, ulcers, warts, etc. [40–41]. Figs have been reported to exhibit antioxidant [34], antibacterial, anti-fungal [42], antispasmodic, antiplatelet [43], antipyretic [44], anti-HSV [45], haemostatic [46], hypoglycemic [47], anticancer [48–49], hepatoprotective [50], antituberculosis [51] and hypo-lipidemic activities [52]. The leaves have been used traditionally in the treatment of jaundice [53]. Figs are an excellent source of phenolic compounds, such as pro-anthocyanidins [54]. Actually, red wine and tea, two well-publicized sources of phenolic compounds contain lower amounts of polyphenols than figs [55]. *Ficus carica* has been reported to excellent the radical scavenging and antioxidant [34] activities.

Fruits of the date palm (Phoenix *dactylifera L*. *Arecaceae*) are commonly consumed in several parts of the world and represent a staple food in most of the Arabian countries. The date fruit has been used in folk remedies for the treatment of various infectious diseases, cancer and immunomodulatory activity [55]. Numerous studies have also shown the antibacterial, antihyperlipidemic activity [56,57], hepatoprotective activity [58], nephroprotective activity [59], Anticancer activity [60], anti-fungal [61,62] properties and antimutagenic activity [63] of date fruits.

Dates are a good source of energy, vitamins, and important elements such as phosphorus, iron, potassium, and a significant amount of calcium [64]. Besides nutritional value, date fruits are rich in phenolic compounds with free radical scavenging and antioxidant activity. Several studies have reported such activities of date fruits cultivated in Algeria [65], Kuwait [63], Oman [66], Iran [67], Bahrain [68] and the USA [65]. These studies showed different amounts of phenolic acids in fresh and dried dates. Studies with three varieties of Omani dates have shown the presence of both free (protocatechuic acid, vanillic acid, syringic acid, and ferulic acid) and bound phenolic acids (gallic acid, protocatechuic acid, p-hydroxybenzoic acid, vanillic acid, caffeic acid, syringic acid, p-coumaric acid, ferulic acid, and coumaric acid) [66]. The potent antioxidant activity of dates is due to its phenolic compounds and flavonoid constituents [69–70]. Date varieties from different regions of Oman had different levels and patterns of phenolic acids. Nine phenolic acids (gallic, protocatechuic, p-hydroxybenzoic, vanillic, caffeic, syringic, p-coumaric, ferulic, and o-coumaric acid) have been tentatively identified. It was found that ferulic acid was the major phenolic acid for all date varieties in Oman [70].

We have recently reported that the dietary supplementation of pomegranates, figs, or date palm fruits growing in Oman can provide benefits against behavior and oxidative stress related abnormalities in an APPSw2576 transgenic mouse model of AD [71–73]. In this study, we examined whether dietary supplementation with pomegranates, figs or date palm fruits can attenuate the levels of AD-like Aβ, inflammatory cytokines and ATP in aged APPsw/Tg2576 mice as an *in vivo* model for AD in comparison to wild type aged mice.

## Materials and Methods

### Collection of Fruits and Diet Preparation

Fresh pomegranates, figs, or date palm fruits were purchased from Al-Jabal Al-Akdhar farms, Oman. All the fruits were frozen at (-40°C) for 5 days. After that, the samples were ground into fine powder using a coffee grinder. The ground fruits were sent to USA to prepare the separate diets for the mice. The diets were prepared by mixing the powdered fruits (4% w/w)[71–76] individually with regular diet as per National Institutes of Health (NIH), USA protocol by Research Diets Inc, NJ, USA.

### Animals and treatment

Sixty four transgenic females (APPsw/Tg2576) and 16 wild-type control (non-transgenic) mice (Taconic Farm, NY, USA) were used for this study. Animals were quarantined for 7 days after shipping and individually housed in plastic cages in an animal room, which was maintained at a temperature of 22±2°C, a relative humidity of 50±10%, and a 12-h light/dark automatic light cycle (light: 0800–2000 h). Tap water was offered *ad libitum* throughout the study. The study was approved by the Animal Care and Use Committee of the Sultan Qaboos University, Oman (SQU/AEC/2010-11/3), and all the procedures involving animals and their care were carried out in accordance with international laws and policies (EEC Council directives 86/609, OJL 358, 1 December, 12, 1987; NIH Guide for the Care and Use of Laboratory Animals, NIH Publications No. 85–23, 1985). All these animals are free from pathogens and viruses. Experimental period commenced from the age of 4 months. The animals were divided into five groups (n = 16/group): Group 1: Wild type (non-transgenic) control of the APPsw mice fed with regular diet, Group 2: AD transgenic mice also fed with regular diet, Group 3: AD mice fed with 4% pomegranates, Group 4: AD mice fed with 4% figs and Group 5: AD mice fed with 4% date fruits. The experimental and control mice were fed an 4% pomegranates, figs, dates, or a control diet for 15 months and then assessed for the effect of each diet on plasma cytokine levels (IL-1β, IL-2, IL-3, IL-4, IL-5, IL-6, IL-9, IL-10, TNF-α and Eotaxin), Aβ and ATP.

### Sample collection

The day after completion of the behavioral tests (data not included), the animals were anesthetized with an intraperitoneal injection of ketamine (75 mg/kg) and xylazine (5 mg/kg), and blood (about 1.5 mL) was collected from the anterior vena cava and placed into heparinized tubes. Collected blood samples were centrifuged at 4000 RPM for 15min at 4°C to obtain the plasma. Brains of experimental and control animals were carefully removed, and homogenized in 9 volumes (1:9 w/v) of cold saline, centrifuged and collected the supernatant. The samples of the brain and plasma were stored at −80°C until measurement.

### Cytokine analyses

The plasma levels of many cytokines were measured using Bio-Rad Bio-Plex kits (Bio-Rad, catalogue # 171F11181). Samples and standards were prepared using the manufacturer’s protocols with the initial concentration of standards ranging from 32 ng/ml to 1.95 pg/ml. Plasma samples were prepared for analysis by diluting 1 volume of the serum sample with three volumes of the Bio-Plex mouse sample diluent. Using the microplate readout, each cytokine level was calculated based on its own standard curve.

### Aβ ELISA analysis

Hippocampal and cortex levels of soluble Aβ1–40 and Aβ1–42 were measured by enzyme-linked immunosorbent assay (ELISA). Briefly, 30 mg brain tissues were homogenized in 400 μL of RIPA buffer [100 mm Tris (pH 8.0), 150 mM NaCl, 0.5% deoxycholic acid, 1% nonyl phenoxylpolyethoxy ethanol-40, 0.2% sodium dodecyl sulfate, and 1 tablet protease inhibitor per 100 mL (S8820; Sigma, St. Louis, MO, USA)], and sonicated for 20 s on ice. Samples were then centrifuged for 30 min at 27,000 x *g* at 4°C, and the supernatants were transferred into new screw cap tubes. The supernatants obtained from this protocol were then stored at -80°C for determination of soluble Aβ levels using ELISA kits (KHB3482 for Aβ1–40, KHB3442 for Aβ-1–42; Invitrogen, Carlsbad, CA, USA). Standards and samples were mixed with detection antibody and loaded onto the antibody-pre-coated plate at the designated wells. After washing the unbound samples, horseradish peroxidase-conjugated antibody was added to all plates, and the substrates of were added for colorimetric reaction, which was stopped with sulfuric acid. Optical density was obtained and concentrations were calculated according to the standard curve.

### Estimation of ATP

ATP contents in mouse brains were determined, as described by Tota et al. [77] using ATP colorimetric/fluorometric assay kit (Biovision, Catalog # K354-100) by following the manufacturer’s instructions.

### Assessment of IL-1β, TNF-α and IL-6

Each brain section was mixed with 10 volumes of ice-cold buffer (20 mM Tris–HCl, pH 7.4) containing 0.5 mM PMSF, 0.5 mM benzamidine, 1.0 mM DTT and 1.0 mM EDTA. Total protein was mechanically dissociated from tissue using an ultrasonic cell disrupter. The sonicated samples were immediately centrifuged at 30,000 × *g* for 30 min at 4°C and the supernatants were removed and stored at 28°C until an ELISA was performed. Total protein concentrations of sonicated brain samples were determined by using a Bio-Rad assay kit using bovine serum albumin as the standard. The ELISAs for mouse IL-1β, TNF-α and IL-6 were performed by using the commercially available kits from Endogen (Woburn, MA, USA).

### Data Analysis

Statistical analysis was performed using the software statistical package SPSS 12 (SPSS, Chicago, IL, USA). A univariate analysis of variance was performed using genotype (wild-type and transgenic), treatment (transgenic + pomegranates), and their interactions as between-individuals fixed factors. According to this, differences between treatments and genotype, or differences between transgenic and treatment were analyzed. Results are provided as mean values ± standard deviation (SD), student’s t test employed. For all tests, the level of statistical significance was set at P < 0.05.

## Results

### Effects of pomegranates, figs, or date palm fruits on plasma cytokine levels

**Fig 1:**
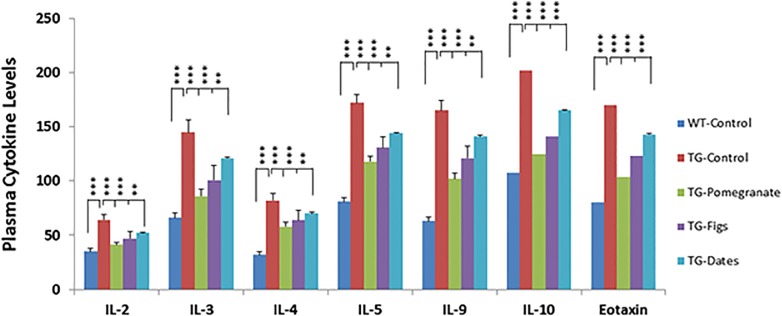
Protective effects of pomegranates, figs, or dates on the plasma levels of many cytokines. The basal plasma levels of many cytokines in WT mice and transgenic mice, fed a control diet or a diet supplemented with 4% total extracts of pomegranates, figs, or dates, as indicated, were determined by multi-plex cytokine anaylsis using the Bio-Plex kits, as described in the Materials and Methods. The representative data from at least three independent experiments are shown (** *p*<0.01 and *** *p*<0.001 vs. control TG group; *n* = 16/group).

As described in Material and Mmethod section, blood plasma from control and experimental animals were used to measure the levels of various cytokines (IL-2, IL-3, IL-4, IL-5, IL-9, IL-10 and Eotaxin). As shown in Fig. 1, the basal levels of these cytokines in control APPsw/Tg2576 mice were significantly increased (0.82 to 1.56 fold) in comparison to those of control wild-type mice. The levels of IL-5 and Eotaxin were almost similar in both control wild type and APPsw/Tg2576 mice. However, the elevated plasma cytokine levels in the APPsw/Tg2576 mice exposed to 4% pomegranates were significantly decreased in comparison to untreated control APPsw/Tg2576 mice (29.30 to 40.64%) followed by figs (22.27 to 30.49%) and dates (14.85 to 18.72%). Among all the cytokines, the total level of IL-10 was highest, ranging from 202.10 ± 15.47 pg/mL, in comparison to IL-2 which was 64.03 ± 4.90 pg/mL. The basal levels of plasma cytokine levels of control wild type were between 63.00 ± 4.80 to 108.02 ± 8.23 pg/mL, respectively with the exception of IL-2, which was 35.01 ± 2.67, and IL-4 which was 32.03 ± 5.03 pg/mL. Among all three fruits tested, significant decrease in plasma cytokine levels was observed in the pomegranate-supplemented diet, suggesting that pomegranate juice is more effective in decreasing pro-inflammatory cytokines and may mediate anti-inflammatory effects. This trend was consistently observed in all other cytokines measured and their amounts were significantly different from those of controls.

### Effect of pomegranates, figs, or date palm fruits on Aβ content in AD model mice

**Fig 2:**
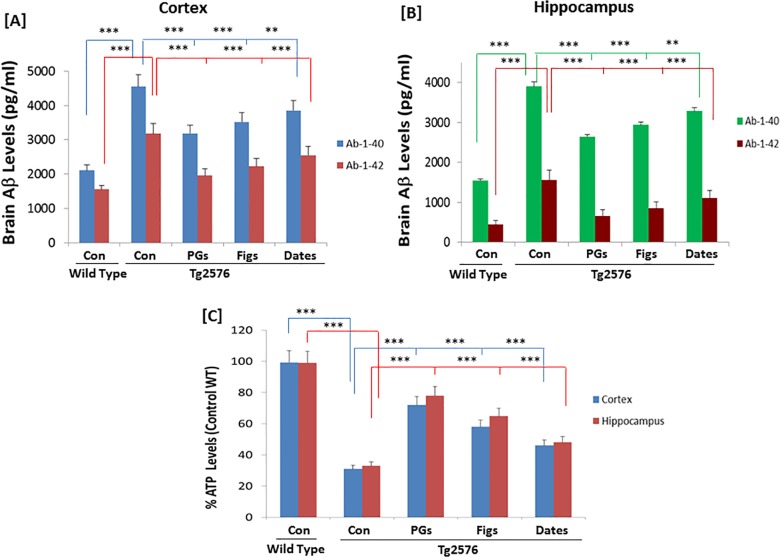
Effects of pomegranates, figs, or dates on the Aβ and ATP levels in the cortex and hippocampus. The levels of Aβ1–40 and Aβ1–42 in the cortex [A] and hippocampus [B] in WT and TG mice, fed a control diet or a diet supplemented with 4% total extracts of pomegranates [PGs], figs, or dates, as indicated, were determined by the ELISA as described in the Materials and Methods. [C] The % ATP levels in the cortex (Blue bars) and hippocampus (red bars) in WT or TG mice fed a control diet or a diet supplemented with pomegranates, figs, or dates are presented. The representative data from at least three independent experiments are shown (** *p*<0.01 and *** *p*<0.001 vs. control TG group; *n* = 16/group).

In brain, the cerebrum or the cortex is the largest portion of the brain and performs most of the brain's function. The cerebrum is divided into right and left hemispheres that are made of nerve cells which are connected by axons carrying the signals between the peripheral organs and the nerve cells. The hippocampus, an elaboration of the edge of the cerebral cortex and located in the cerebral hemisphere, is responsible for learning and memory, specifically converting temporary memories into permanent memories. These represent some regions of the brain that are susceptible to damage in neurodegenerative diseases.After measuring the levels of plasma cytokine levels, we next determined the levels of Aβ1–40 and Aβ1–42, since the accumulation of Aβ peptides activate neuro-inflammation in AD. Brain samples were collected from the untreated control or animals supplemented with pomegranates, figs or dates. The basal levels of Aβ1–40 in the cortex of control WT mice were 2105.35 ± 160.31 pg/mL and 1.36-fold higher than hippocampus (1540.26 ± 117.28 pg/mL) (Fig. 2A). Similarly, the basal levels of Aβ1–42 in the cortex of control wild-type was 1524.21 ± 95.50 pg/mL and 3.43-fold higher than hippocampus (452.08 ± 34.42 pg/mL) (Fig. 2A). The levels of Aβ1–40 in control TG (APPsw/Tg2576) were 4552.28 ± 348.45 pg/mL, which is significantly higher than that of control wild type (3906.95 ± 299.05 pg/mL) (Fig. 2A & B). The levels of Aβ1–40 in the brains of animals supplemented with the pomegranate diet decreased significantly (30.01% and 32.24%) in the cortex and hippocampus, respectively. The Aβ1–42 levels were 1956.33 ± 148.97 and 658.11 ± 50.11 pg/mL in the cortex and hippocampus, respectively. These levels in cortex and hippocampus were significantly decreased by 38.50% and 57.88% respectively (Fig. 2A & B). Supplementation of pomegranates significantly reduced the levels of Aβ1–40 and Aβ1–42 in APPsw/Tg2576 mice. This degrees of suppression of Aβ peptide levels were also observed in animals fed diets with figs or dates. The levels of Aβ1–40 in the cortex and the hippocampus in APPsw/Tg2576 mice were high and the levels of Aβ1–42 in the cortex, were ∼25–40% less than that of Aβ1–40. Interestingly, the levels of Aβ1–42 in hippocampus were estimated to be ∼60–70% less than the levels of Aβ1–40, suggesting that the Aβ1–40 peptide is the major aggregated peptide observed in AD.

### Effect of pomegranates, figs, or date palm fruits on brain ATP content of mice brains

After measuring the levels of Aβ1–40 and Aβ1–42 which serve as the biomarkers for AD, while they deplete the total ATP levels in the brain. ATP is required for numerous metabolic activities and neuronal survival. The decreased ATP levels may affect normal neuronal functions and may promote pro-inflammatory conditions by activating microglia and releasing prostanoids. Hence, to measure the ATP levels, the cortex and hippocampal regions of the brains from different groups were isolated as described in Materials and Methods. The levels of ATP in control TG mice decreased significantly (∼70%) in comparison to control wild type (Fig. 2C). However, when the animals were fed with pomegranates, figs, or dates containing diets, we observed significant recovery in ATP levels. The recovery of ATP levels ranged from 78% as observed in pomegranates followed by ∼60% (figs and ∼45% (dates), respectively. These results suggested that pomegranates could improve cerebral energy production in AD (APPsw/Tg2576) mouse model caused by aggregation of Aβ peptides (Fig. 2C).

### Effect of pomegranates, figs and date palm fruits on brain IL-1β, TNF-α and IL-6 levels

**Fig 3:**
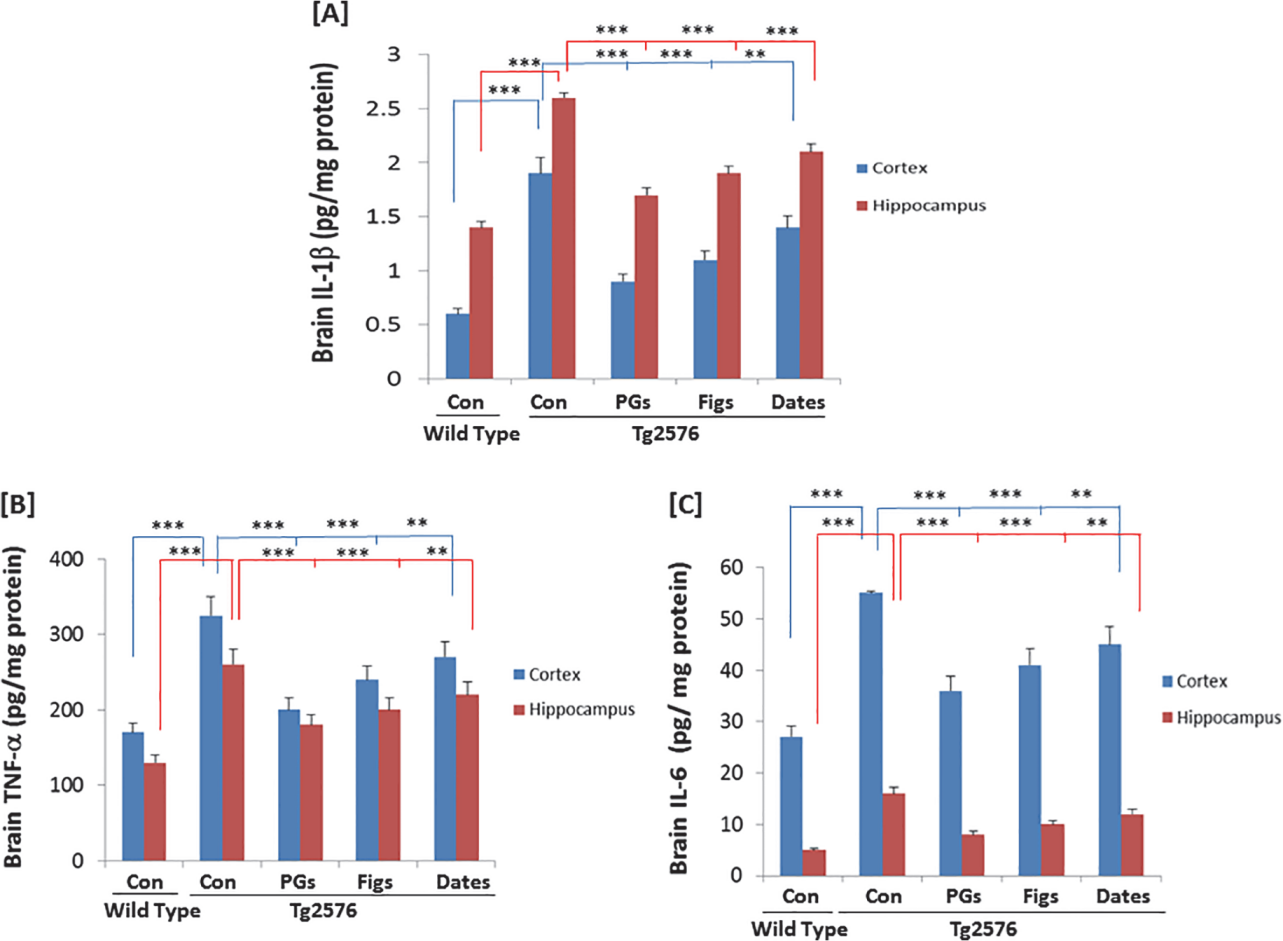
Effects of pomegranates, figs, or dates on the levels of IL-1β TNF-α and IL-6 in the cortex and hippocampus. The levels of the three cytokinses in the WT and TG mice, as indicated, were determined by multi-plex cytokine anaylsis using the Bio-Plex kits, as described in the Materials and Methods. The representative data from at least three independent experiments are shown (** *p*<0.01 and *** *p*<0.001 vs. control TG group; *n* = 16/group), Pomegranates [PGs].

After determining the levels of pro-inflammatory cytokines in the blood plasma, we measured the levels of cytokines in the brain regions, particularly in the cortex and hippocampus (Fig. 3A). Since some of the cytokines, for instance IL-6, may act as both pro- and anti-inflammatory agents, we measured the cellular levels of these cytokines including TNF-α and IL-1β. The basal levels of IL-1β in the hippocampal region were 1.40 ± 0.06 pg/mg protein the amounts in cortex were 0.60 ± 0.05 pg/mg protein, indicating about 2.33-fold greater amount in the hippocampus than that in the cortical region. However, in control APPsw (Tg2576) mice, the levels of IL-1β increased significantly, reaching 1.85 and 3.16 times greater than the basal protein levels in the cortex and hippocampus, respectively. When the animals were fed with pomegranate supplemented diet, the levels of IL-1β decreased 1.21 and 1.50 times in cortex and hippocampus, respectively, suggesting that pomegranates and other fruit supplementation could indeed reduce the levels of IL-1β and decrease neuroinflammatory activities. A similar trend was observed in the experimental animals that were fed with figs, or dates (Fig. 3A). However, the protective effects of pomegranates were more prominent, and followed by diets supplemented with figs or dates. The cellular levels of TNF-α and IL-6 in both cortex and hippocampus in Tg mice were higher as compared to those in wild control mice (Fig. 3B & C). However, the elevated TNF-α and IL-6 levels in both cortex and hippocampus in Tg mice were suppressed after the Tg mice were fed with diets supplemented with pomegranates, figs, or dates.

## Discussion

Many experimental animal models for AD are available to study the pathogenesis mechanisms and translational research. For instance, the proposed model for neurodegeneration in AD brains is based on free radical mediated oxidative stress associated with Aβ1–40 and Aβ1–42 accumulation [78–79]. The role of Met-35 as a mediator of the toxicity of Aβ is more likely to involve an oxidative event at the sulfur atom, leading to lipid peroxidation and protein oxidation in neuronal membranes. However, the event that initiates the oxidation of Met-35 is not yet clear. The increased levels of Aβ in AD have been shown [80–81]. Furthermore, in an experimental mouse model of AD,greater amounts of Aβ1–42 and Aβ1–40 are started to be secreted after a few months of age and then accumulated in Tg2576 mice than their wild type control litter mates throughout their lives [82–83]. By using the Tg2576 mouse model for AD, we aimed to study the beneficial effects of antioxidants present in pomegranates, figs, or dates on a few neuro-inflammatory markers in blood plasma and brain regions. For this purpose, we specifically selected the fruits that are grown in Oman. Many studies suggest that different species from various geographical areas have diverse micronutrients and other bioactive components that may prevent or alleviate pathophysiological conditions. Our results showed significant increase in Aβ1–40 and Aβ1–42 levels both in the cortex and hippocampus. These results, consistent with the already published reports, suggest that the increased Aβ1–40 and Aβ1–42 accumulation is likely to promote oxidative stress responsible for the progression of neurodegeneration. Extended supplementation with pomegranates, figs, or dates (for 15 months) indeed decreased the Aβ1–40 and Aβ1–42 levels in the brain of Tg2576 mice in comparison to control diet-fed mice. Despite being small but significant, the observed decreases are promising, when considering the dynamic nature of Aβ present in plasma samples. Our previous studies demonstrated that dietary supplementation with pomegranates attenuates cognitive and behavioral deficits in a transgenic mouse model of AD [84].

The roles of pro-inflammatory cytokines in mediating a number of metabolic and neurological diseases are well documented. Our current results showed that the levels of pro-inflammatory cytokines, particularly IL-1β, TNF-α and IL-6 in the brains of experimental animals increased in APPsw (Tg2576) mice. The levels of IL-1β, TNF-α and IL-6 in the cerebral cortex and the hippocampus were decreased in the brains of Tg2576 mice fed diets supplemented with pomegranates, figs or dates. IL-1β, a critical cytokine in the orchestration of the complex immune response to infection and injury [85], was originally described as a peripheral immune cell mediator. This cytokine has also been reported to be synthesized in the brain by glial cells and certain neurons; and IL-1β receptors have been found in different regions of the brain, with the highest abundance in the hippocampus [86–88]. Proinflammatory cytokines including IL-1β, TNF-α and IL-6 have been reported to be significantly elevated in the cerebro-spinal fluid or plasma of AD patients [89–90]. In addition, a important role of inflammation in AD is well-supported through the inverse relationship between anti-inflammatory drug therapy and the onset and symptoms [91–92]. Griffin et al [93], reported the expression of IL-1β in different plaque types in AD, indicating that an inflammatory response plays a central role in plaque development and dystrophic neurite formation. IL-6 was present during the early stages of plaque formation and expression of this cytokine was correlated with clinical dementia [94]. IL-1β augments Aβ-peptide cytotoxicity in rat pheochromocytoma cells [95]. It has been reported that β-amyloid proteins and interferon (IFN)-δ activate microglia to produce neurotoxic TNF-α and reactive nitrogen intermediates and these events may play a role in the pathogenesis of neuronal degeneration observed in aging and AD [96].

The mechanism of the reduction of IL-1β, TNF-α and IL-6 by pomegranates is uncertain, since its multiple active components such as anthocyanins, ascorbic acid, ellagic acid, gallic acid, fumaric acid, caffeic acid, catechin, EGCG, quercetin, rutin, tannins, alkaloids and flavanoids, have multifunctional action, thus making it pharmacologically complex. Our current results, in agreement with previous reports, suggest that pomegranates in diet indeed decreased the cytokine levels [97–102]. However, the antioxidant properties of pomegranates have been well-documented. These properties include free radical scavenging and inhibition of lipid peroxidation as well as enhancement of antioxidant status [103–105] and neuroprotection [28,106–108].

Similarly, the date palm fruits also contain flavonoid glycosides of luteolin, quercetin, and apigenin. Recent research studies suggest that apigenin exhibits some mild sedative effects with anti-inflammatory properties [109–110] and neuroprotection.

Many fruits, when compared to vegetables and cereals, have very high anti-oxidant values, which are measured in terms of their "Oxygen Radical Absorbent Capacity" or ORAC. These compounds have potent anti-oxidant properties that help remove free radicals from the body, and thus provide protection against cancers, aging, and neurodegeneration. All these compounds help the body prevent or at least prolong the natural changes of aging by protecting from damage and rejuvenating cells, tissues, and organs. Including fruits in the daily diet protects from minor ailments like wrinkling of skin, hair-fall, and memory loss to major ailments like age-related macular degeneration of the retina in the eyes, neurodegenerative diseases including AD, cancers, osteoporosis [111]. Research supporting the beneficial roles of phytochemicals against cancers, coronary heart disease, diabetes, high blood pressure, inflammation, microbial, viral and parasitic infections, psychotic diseases, spasmodic conditions, ulcers, etc is based on the chemical mechanisms using *in vitro* cell culture systems, various disease states in animals and the epidemiology of humans [112].

## Conclusion

Natural fruits, nuts, herbs and vegetables constitute a wide array of biologically active compounds including ferulic acid, anthocyanins, ellagic acid, punicagins, flavonols, phenolic acids and very important micronutrients such as phosphorus, iron, potassium and calcium, that are found abundantly in the plant kingdom. They are gaining interest due to their beneficial properties and with minimum side effects. Some of these natural products are effective in treating or preventing the majority of cardiovascular, metabolic and neurodegenerative diseases. Antioxidant activity is the key factor of all flavonoids by which they mediate the beneficial effects against the majority of many different diseases. The actions of dietary flavonoids involve a number of effects within the brain, such as modulation of neuronal signaling and the protection against neuronal losses. An extensive study on structure-function relationships of flavonoid activities provides valuable information for rationale drug designs of future pharmaceuticals in the prevention and treatment of several life-threatening diseases.

**Fig 4:**
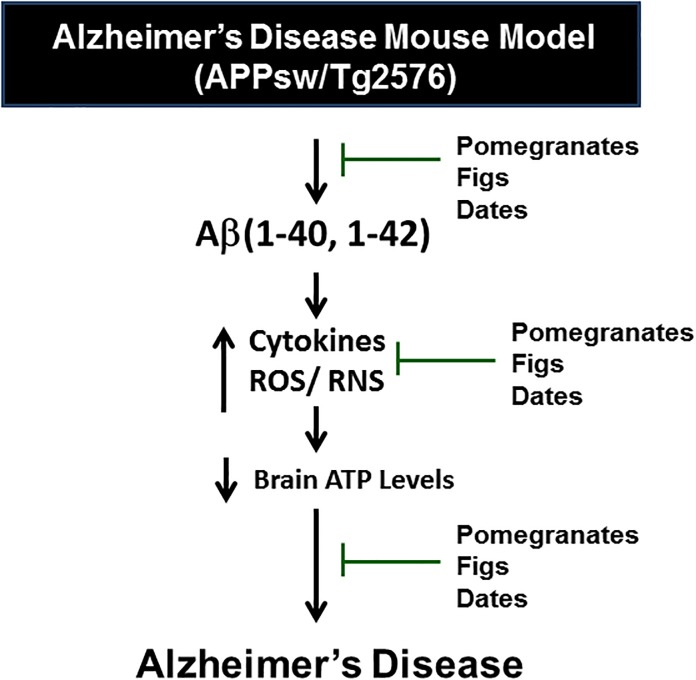
Schematic diagram. The conclusive figure showing the inflammatory signaling pathways in Alzheimer’s disease mouse model (APPsw/Tg 2576) and protection by dietary supplementation of pomegranates, figs and dates as potential complementary and alternative medicine for the neurodegenerative diseases.

In conclusion, the pomegranates, figs, and date palm fruits grown in Oman provide possible protection against the inflammation in Tg2576 AD mouse brain and the mechanisms of protection may be related to their antioxidant activities of phenolic constituents (Fig. 4). Based on the *in vivo* experimental studies and the active ingredient profiles, it can be concluded that these fruits showed promising therapeutic potential against neurodegenerative diseases including AD, that areassociated with elevated inflammation. However, these results warrant further investigation of the mechanisms by which anti-inflammatory properties of these fruits can exert such beneficial effects on the brain in AD-like models.

## Supporting Information

S1 Dataset The data set and supporting informations for Fig. 1. (XLSX)

S2 Dataset The data set and supporting informations for Figs. 2 and 3. (XLSX)
